# Asymmetric Large Kernel Distillation Network for efficient single image super-resolution

**DOI:** 10.3389/fnins.2024.1502499

**Published:** 2024-11-11

**Authors:** Daokuan Qu, Yuyao Ke

**Affiliations:** ^1^School of Information and Control Engineering, China University of Mining and Technology, Xuzhou, Jiangsu, China; ^2^School of Energy and Materials Engineering, Shandong Polytechnic College, Jining, Shandong, China; ^3^School of Computer Science and Technology, China University of Mining and Technology, Xuzhou, Jiangsu, China

**Keywords:** single image super-resolution, efficient method, asymmetric large kernel convolution, information distillation, convolutional neural network

## Abstract

Recently, significant advancements have been made in the field of efficient single-image super-resolution, primarily driven by the innovative concept of information distillation. This method adeptly leverages multi-level features to facilitate high-resolution image reconstruction, allowing for enhanced detail and clarity. However, many existing approaches predominantly emphasize the enhancement of distilled features, often overlooking the critical aspect of improving the feature extraction capabilities of the distillation module itself. In this paper, we address this limitation by introducing an asymmetric large-kernel convolution design. By increasing the size of the convolution kernel, we expand the receptive field, which enables the model to more effectively capture long-range dependencies among image pixels. This enhancement significantly improves the model's perceptual ability, leading to more accurate reconstructions. To maintain a manageable level of model complexity, we adopt a lightweight architecture that employs asymmetric convolution techniques. Building on this foundation, we propose the Lightweight Asymmetric Large Kernel Distillation Network (ALKDNet). Comprehensive experiments conducted on five widely recognized benchmark datasets-Set5, Set14, BSD100, Urban100, and Manga109-indicate that ALKDNet not only preserves efficiency but also demonstrates performance enhancements relative to existing super-resolution methods. The average PSNR and SSIM values show improvements of 0.10 dB and 0.0013, respectively, thereby achieving state-of-the art performance.

## 1 Introduction

Single image super-resolution (SISR) is a fundamental task in low-level computer vision, aimed at recovering fine details lost during image degradation and reconstructing a high-resolution (HR) image from a given low-resolution (LR) input. In recent years, the advancement of deep learning has led to numerous methods leveraging deep neural networks to address the challenges of image SR.

Dong et al. (2014) were the first to apply convolutional neural networks to image SR. Their method involved upsampling the low-resolution image to match the high-resolution size using bicubic interpolation, followed by the use of a Super-Resolution Convolutional Neural Network (SRCNN) to learn the mapping from the upsampled image to the high-resolution counterpart. Although SRCNN consisted of only three convolutional layers, it achieved remarkable performance. Kim et al. (2016a) introduced residual connections in their Very Deep Super-Resolution (VDSR) network, which enabled deeper networks (up to 20 layers) and significantly improved reconstruction performance. In response to the limitations of residual networks for low-level vision tasks, Lim et al. (2017) proposed the Enhanced Deep Super-Resolution (EDSR) network, which utilized simplified residual blocks by removing redundant batch normalization layers. Their findings demonstrated that batch normalization was unnecessary for SR tasks, leading to fewer reconstruction artifacts and reducing the computational complexity of the model. Nevertheless, the reliance of these super-resolution methods on intricate deep convolutional neural networks poses significant challenges for practical deployment, particularly in resource-constrained settings such as real-time processing, mobile platforms, or embedded devices.

Various methods have been introduced to address lightweight SR task, including recurrent learning (Kim et al., 2016b), neural network pruning (Zhang et al., 2021a,b; Wang et al., 2023), knowledge distillation (Gao et al., 2018; He et al., 2020), neural architecture search (Chu et al., 2021), etc. Recently, information distillation (Hui et al., 2018) has emerged as a preferred strategy for designing lightweight networks for super-resolution. This technique involves stacking distillation blocks, which incorporate feature enhancement and compression units, to extract features at different depths for image reconstruction. IMDN (Hui et al., 2019) expands on the concept of information distillation by employing a distillation module and a fusion module within each Information Multi-Distillation Block (IMDB) to extract and integrate hierarchical features. Building on this foundation, RFDN (Liu et al., 2020) introduces a shallow residual block that enhances performance without increasing the number of parameters. BSRN (Li et al., 2022) employs Blueprint Separable Convolutions (BSConv) (Haase and Amthor, 2020) to optimize the Super Resolution Block (SRB) and integrates enhanced spatial attention for feature refinement, achieving state-of-the-art results. BSConv operates on the premise that a blueprint serves as a template for the convolutional weights, allowing all convolution kernels within a model to be derived through linear transformations of this blueprint. Specifically, BSConv first performs a weighted combination of depth features, followed by channel-wise convolutions to regulate the interdependencies within the learned convolution kernels. However, this regulation inadvertently limits the potential for further enhancement in feature extraction capacity.

To address this issue, we present an Asymmetric Large Kernel Distillation Network (ALKDNet), designed to enhance the quality of reconstructed images while maintaining efficient super-resolution performance. The proposed method incorporates large kernel convolutions to better extract and refine features. Increasing the kernel size effectively expands the receptive field, allowing the model to leverage more contextual information for improved task completion. However, directly enlarging the kernel size leads to a dramatic increase in parameters and computational cost. To mitigate this, we propose an asymmetric large kernel convolution, which replicates the effects of a large kernel by utilizing two asymmetric rectangular convolutions and a smaller square convolution. Additionally, we introduced an Anchor-Based Residual Learning (ABRL) (Du et al., 2021) method, built upon the conventional feature space residual learning, to further enhance the visual quality of the reconstructed images. This method establishes anchor points for each pixel in the high-resolution image using the corresponding low-resolution pixels, providing richer detail for image reconstruction.

Our contributions in this paper can be summarized as follows:

- We propose a novel Asymmetric Large Kernel Distillation Network (ALKDNet) aimed at addressing the challenge of lightweight super-resolution. Experiments on benchmark datasets demonstrate that the proposed ALKDNet achieves state-of-the-art performance.
- We design a novel Asymmetric Large Kernel Convolution (ALKConv), which enhances model performance while preserving computational efficiency and manageable complexity.
- We incorporate an anchor-based residual learning method into our ALKDNet alongside the conventional feature space residual learning, which results in improved performance compared to using either residual learning method in isolation.

The remainder of the paper is organized as follows: Section 2 shows an overview of the related work, Section 3 details the proposed model, Section 4 presents the empirical research results, and Section 5 shows the conclusion.

## 2 Related work

### 2.1 Efficient SR methods

As previously mentioned, Dong et al. (2014) were the first to apply CNNs to the SR problem, though their initial method was highly inefficient. In response, they introduced FSRCNN (Dong et al., 2016), which utilized a deconvolution layer as the upsampling module placed at the end of the network. This significantly accelerated the model and established a new paradigm for network design in SR tasks. Subsequently, ESPCN (Shi et al., 2016) proposed a sub-pixel convolutional upsampling method that delivered superior performance, making it the go-to upsampling strategy for SR tasks. Kim et al. (2016b) introduced recursive learning in DRCN, reducing the model size without sacrificing effectiveness. Subsequently, Tai et al. (2017) enhanced DRCN by proposing the Deep Recurrent Residual Network (DRRN), which achieved superior performance with fewer parameters while maintaining the same network depth. Building upon the Laplacian pyramid framework, Lai et al. (2017) developed a deep laplacian pyramid network (LapSRN), which leverages low-resolution feature maps at each pyramid layer to predict high-frequency details, achieving notable performance improvements. Ahn et al. (2018) advanced this by proposing CARN, which incorporated a cascading mechanism into the residual network. Hui et al. (2018) were the first to apply the information distillation mechanism for efficient SR in their IDN. Later, Hui et al. (2019) extended this concept with IMDN, introducing information multi–distillation, which considerably boosted model performance. RFDN (Liu et al., 2020) further lightened the model while improving its performance by designing shallow residual blocks and incorporating extensive feature distillation connections. Finally, BSRN (Li et al., 2022) achieved state-of-the-art results by replacing standard convolutions with blueprint separable convolutions and enhancing feature extraction through enhanced spatial attention, further reducing model complexity. Furthermore, Hui et al. (2020) integrated non-local operations into the residual block architecture, introducing a lightweight Feature Enhancement Residual Network (FERN). This design significantly strengthened the model's capacity to capture long-range dependencies. Moreover, Wang et al. (2021) developed a Sparse Masked Super-Resolution (SMSR) model that utilizes sparse masks. This method employs spatial masks to identify salient regions and channel masks to filter out unnecessary channels, thereby reducing redundant computations and enhancing super-resolution performance. Kong et al. (2022) streamlined the feature aggregation process by employing three convolutional layers for local feature learning, and introduced a Residual Local Feature Network (RLFN), achieving a balance between model performance and inference time. Additionally, Gendy et al. (2023) further advanced the SISR task by proposing a Mixer-based Local Residual Network (MLRN), which utilizes convolutional mixer blocks to blend channel and spatial features, achieving favorable performance.

### 2.2 Large kernel convolution

Since VGG (Simonyan and Zisserman, 2014) popularized the method of replacing large convolution kernels with stacked smaller convolutions, it has been widely adopted for its lightweight and efficient characteristics. With the advent of Transformer (Vaswani, 2017), many researchers sought to understand the source of their superior performance. Some attributed this to the extensive receptive field provided by the attention mechanism and aimed to enhance CNNs by expanding their receptive fields. According to the theory of effective receptive fields (ERF) (Luo et al., 2016), the ERF is proportional to $O\left(K\sqrt{L}\right)$, where *K* represents the kernel size and *L* the network depth. This shows that increasing the kernel size is a more effective way to expand the ERF than merely stacking smaller convolutions. ConvNeXt (Liu Z. et al., 2022) expands the convolution kernel size to enhance the receptive field, ultimately achieving performance comparable to that of the Swin Transformer (Liu et al., 2021). RepLKNet (Ding et al., 2022) leveraged reparameterization technique and depth-wise convolution to scale the kernel size up to 31 × 31, achieving results that are comparable to, and in some cases surpass, those of the Swin Transformer across various tasks. Guo et al. (2023) integrated large kernel convolution with an attention mechanism, introducing a novel Large Kernel Attention (LKA) module in their VAN architecture, which demonstrated significant effectiveness across various tasks. LargeKernel3D (Chen et al., 2023) applied the concept of large kernel design to 3D networks, expanding the kernel size to 17 × 17 × 17. SLaK (Liu S. et al., 2022) simulated large kernel convolutions with two rectangular convolutions and integrated dynamic sparsity, pushing the kernel size to 51 × 51. Meanwhile, PeLK (Chen et al., 2024) further extended the kernel to 101 × 101 using a parameter-sharing mechanism and kernel-based position embedding, achieving impressive results across various computer vision tasks.

### 2.3 Asymmetric convolution

Szegedy et al. (2016) first introduced the concept of asymmetric convolution decomposition in Inception-v3, wherein the 7 × 7 convolution kernel is split into two smaller kernels of 7 × 1 and 1 × 7 to reduce the parameters for image recognition. This technique was adopted in Global Convolutional Network (GCN) (Peng et al., 2017) to increase the kernel size to 15 × 15, enhancing performance in semantic segmentation tasks. However, it has been reported that this method may lead to a decrease in performance on ImageNet. EDANet (Lo et al., 2019) also employed this strategy by substituting 3 × 3 convolutions with 3 × 1 and 1 × 3 convolutions to reduce computational cost, albeit at the expense of performance. Nevertheless, it experienced a decline in performance when applied to semantic segmentation tasks. In contrast, Ding et al. (2019) utilized asymmetric convolution for structural reparameterization in ACNet, where asymmetric convolutions were employed to strengthen horizontal and vertical information, which was then aggregated on a square convolution kernel, leading to significant performance improvements. Furthermore, Tian et al. (2021) were the first to apply asymmetric convolution in the realm of image super-resolution, achieving notable results. Building on this foundation, SLaK (Liu S. et al., 2022) integrates convolution decomposition with dynamic sparsity, expanding the kernel size to 51 × 51 and thereby significantly improving model performance.

## 3 Proposed method

In this section, we firstly introduce the overall network architecture of ALKDNet and the loss function, then we give a detailed introduction to the designed asymmetric large kernel distillation block. Next, we introduce the proposed asymmetric large kernel convolution in detail.

### 3.1 Network architecture

**Figure 1:**
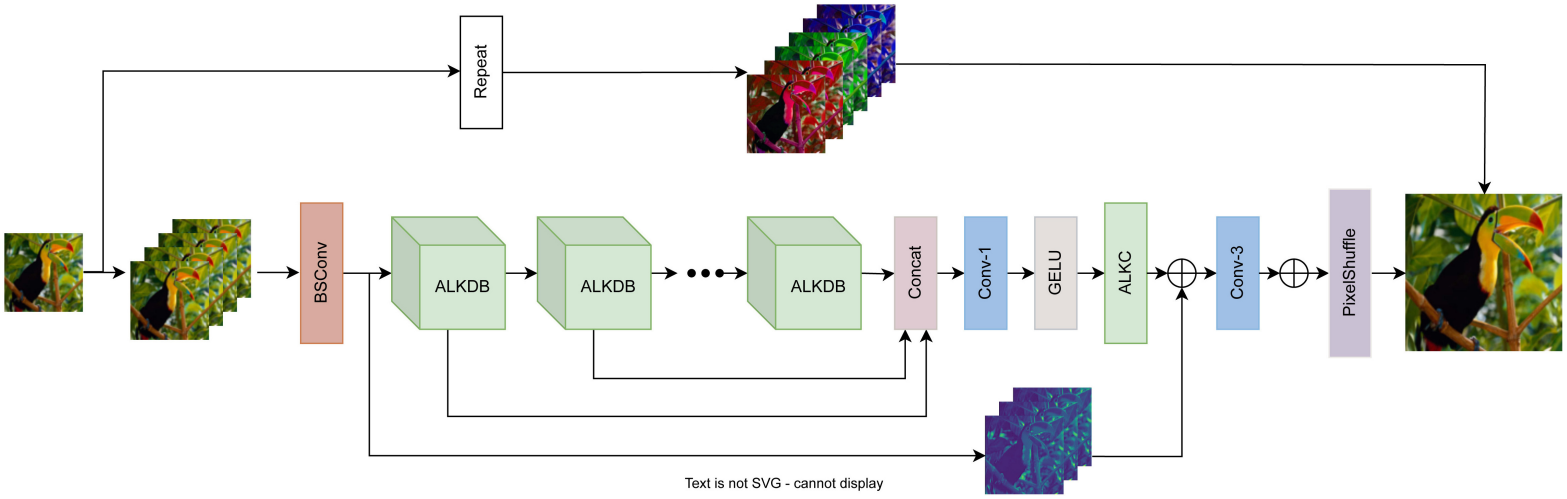
The architecture of ALKDNet. The ALKDNet replicates the LR image *m* times as the input, maps it to the feature space via BSConv, and progressively refines the features through a series of ALKDB blocks. After feature fusion stage, the fused features are sent to the image reconstruction module to obtain the reconstructed image.

The proposed method adopts the structural design of BSRN (Li et al., 2022), as illustrated in Figure 1. The complete model consists of four main components: a shallow feature extraction module, a deep feature extraction module, a deep feature fusion module, and a high-resolution image reconstruction module.

**Figure 2:**
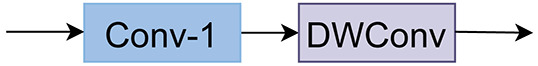
The architecture of BSConv. BSConv initially applies a 1 × 1 pointwise convolution, which is subsequently followed by independent depthwise convolutions executed for each individual channel.

Initially, the input image *I*_*LR*_ is duplicated *m* times and concatenated along the channel dimension to form $I_{LR}^{m}$. This process is described as follows:


(1) $$\begin{array}{l} I_{LR}^{m}=Concat_{m}\left(I_{LR}\right), \end{array}$$

where *Concat*(·) represents the concatenation operation along the channel dimension, where *m* indicates the number of times the input image *I*_*LR*_ is replicated and concatenated. Subsequently, higher-dimensional shallow features are extracted through the shallow feature extraction module:


(2) $$\begin{array}{l} F_{0}=H_{SFE}\left(I_{LR}^{m}\right), \end{array}$$

where *H*_*SFE*_(·) represents the shallow feature extraction module, implemented as a 3 × 3 BSConv, with *F*_0_ denoting the extracted shallow features. The structure of BSConv, illustrated in Figure 2, consists of both a channel convolution and a depthwise convolution. Following this, a series of asymmetric large kernel distillation blocks (ALKDB) are employed to progressively extract and refine deep features. This process can be expressed as follows:


(3) $$\begin{array}{l} F_{k}=H_{k}\left(F_{k-1}\right),k=1,2,...,n, \end{array}$$

where *H*_*k*_ represents the *i*-th ALKDB, while *F*_*k*_ and *F*_*k*−1_ refer to the output and input of the *i*-th ALKDB, respectively.

After the progressive extraction and refinement of ALKDBs, all intermediate features are concatenated via a 1 × 1 convolution, followed by GELU activation for feature fusion and activation. Finally, asymmetric Large Kernel Convolution (ALKConv) is applied to smooth the features. This deep feature fusion process can be described as follows:


(4) $$\begin{array}{l} F_{fused}=H_{fusion}\left(Concat\left(F_{1},...,F_{k}\right)\right), \end{array}$$

where *F*_*fused*_ represents the aggregated deep features, while *H*_*fusion*_ refers to the feature fusion module as described above.

In the final stage, the image reconstruction module of BSRN employs a long-range skip connection for residual learning. While maintaining this residual learning in the feature space, we introduce an anchor-based residual learning method. This method repeats the squared upscaling factor for each pixel in the LR space, using it as an anchor point for the corresponding pixel in the HR space. Subsequently, the pixel shuffle operation is applied to generate the reconstructed image. This process can be formulated as follows:


(5) $$\begin{array}{l} I_{SR}=H_{PS}\left(Conv_{up}\left(F_{fused}+f_{0}\right)+H_{repeat}\left(I_{LR}\right)\right), \end{array}$$

where *H*_*PS*_(·) denotes the pixel shuffle operation, while *H*_*repeat*_(·) refers to repeating the squared upscaling factor of the LR images, organizing them by color channels, and concatenating them along the channel dimension. The *Conv*_*up*_(·) operation is a 3 × 3 convolution, used to expand the fused features learned through residual learning in the feature space, ensuring that their channels are aligned with the output of *H*_*repeat*_(·).

Our model is optimized using the L1 loss function, which is formulated as:


(6) $$\begin{array}{l} L_{1}=||I_{SR}-I_{HR}|{|}_{1}. \end{array}$$

### 3.2 Asymmetric large kernel distillation block

**Figure 3:**
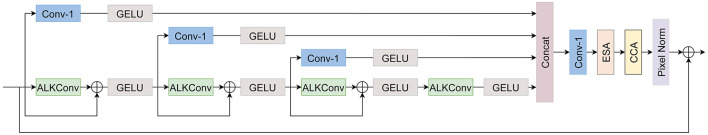
The architecture of ALKDB. ALKDB extracts and refines features using a sequence of ALKConvs, while simultaneously employing 1 × 1 convolutions for feature compression. Following the concatenation and fusion of the features, the ESA and CCA modules are utilized for further feature enhancement.

Drawing inspiration from the ESDB structure in BSRN (Li et al., 2022), we designed a asymmetric large kernel distillation block (ALKDB) with a similar architecture. The ALKDB is composed of three key components: feature distillation, feature condensation, and feature enhancement. The overall structure of ALKDB is illustrated in Figure 3. Given an input feature *F*_*in*_, the feature distillation process in the initial stage can be formulated as follows:


(7) $$\begin{array}{l} F_{d1},F_{r1}=D_{1}(F_{in}),R_{1}(F_{in}),\\ F_{d2},F_{r2}=D_{2}(F_{r1}),R_{2}(F_{r1}),\\ F_{d3},F_{r3}=D_{3}(F_{r2}),R_{3}(F_{r2}),\\ \;F_{d4}=D_{4}(F_{r3}), \end{array}$$

where *D*_*i*_ represents the *i*-th distillation layer, responsible for extracting the distilled feature *F*_*di*_, while *R*_*i*_ denotes the *i*-th refinement layer, used to iteratively refine the feature *F*_*ri*_. Specifically, the distillation layer is composed of a 1 × 1 convolution followed by GELU activation, while the refinement layer consists of a asymmetric large kernel convolution with skip connections, also followed by GELU activation. In the feature condensation stage, the four distilled features are concatenated along the channel dimension, followed by a 1 × 1 convolution for feature fusion. This process can be described as follows:


(8) $$\begin{array}{l} F_{condensed}=Conv_{\text{\_}1}\left(Concat\left(F_{d1},...,F_{d4}\right)\right), \end{array}$$

where *F*_*condensed*_ represents the condensed feature obtained from the fusion process. In the subsequent feature enhancement stage, we employ both a Enhanced Spatial Attention (ESA) block and a Contrast-aware Channel Attention (CCA) block to further enhance the features. Simultaneously, the pixel normalization module is applied to ensure stability during the model's training process:


(9) $$\begin{array}{l} F_{enhanced}=Norm_{pixel}\left(H_{CCA}\left(H_{ESA}\left(F_{condensed}\right)\right)\right), \end{array}$$

where *H*_*CCA*_(·) and *H*_*ESA*_(·) represent the CCA and ESA modules, respectively, while *Norm*_*pixel*_(·) denotes the pixel-level normalization module. The output, *F*_*enhanced*_, is the enhanced feature. Ultimately, the input features *F*_*in*_ are employed for long-range residual learning to derive the final output features *F*_*out*_:


(10) $$\begin{array}{l} F_{out}=F_{enhanced}+F_{in}. \end{array}$$

### 3.3 Asymmetric large kernel convolution

**Figure 4:**
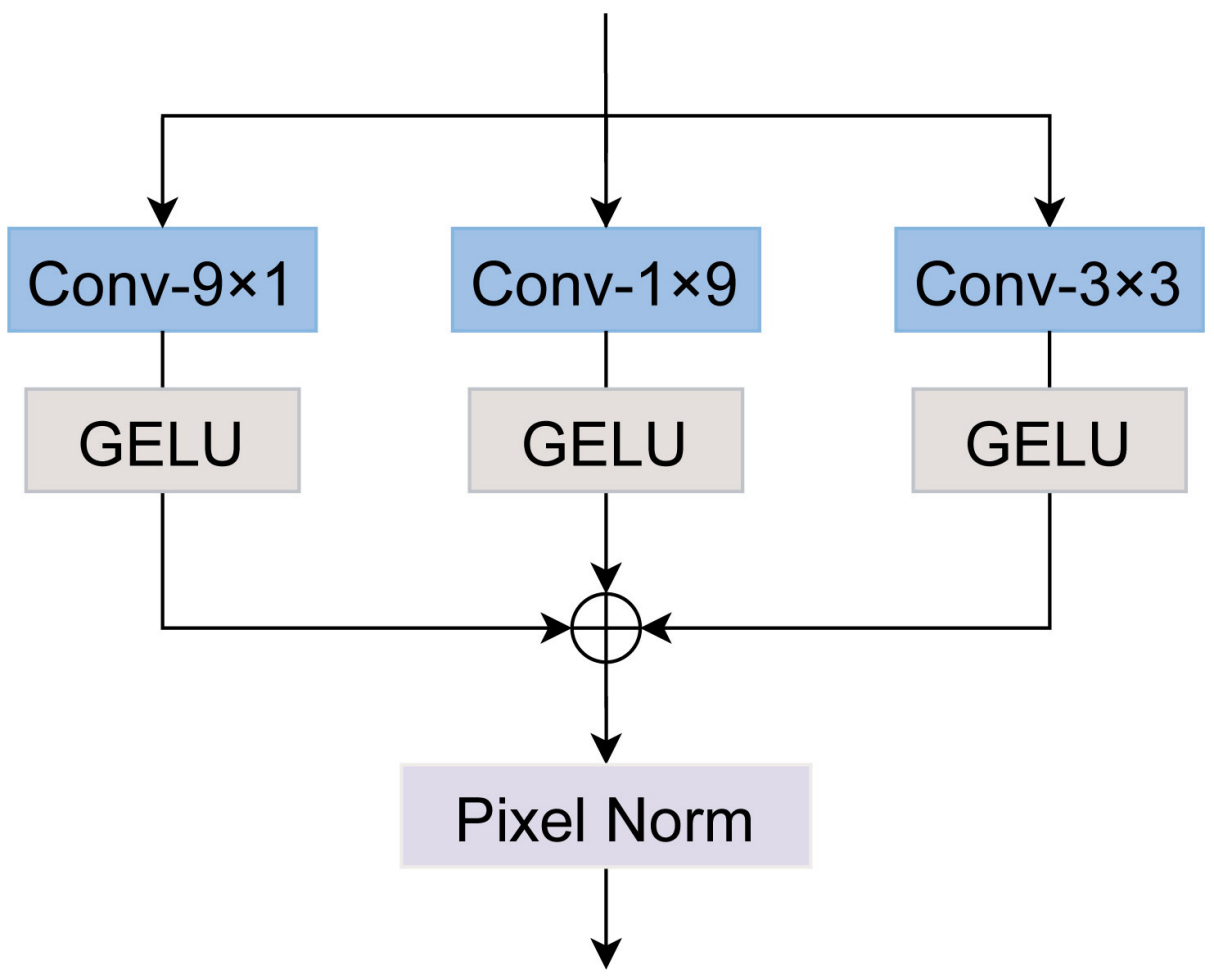
The architecture of ALKConv. We decompose a 9 × 9 convolution into 9 × 1 and 1 × 9 convolutions. In line with prior research, we also introduce a 3 × 3 convolution to operate in parallel with the large kernel convolution, subsequently summing their outputs.

Liu S. et al. (2022) proposed the decomposition of a large 51 × 51 convolutional kernel into three smaller kernels of size 51 × 5, 5 × 51, and 5 × 5 in their SLaK model, enhancing performance while keeping computational complexity manageable. Drawing inspiration from this method, we adopt a similar strategy to construct a 9 × 9 large kernel convolution, as illustrated in Figure 4.

Specifically, for the input feature *F*_*in*_, we apply three convolution operations with kernel sizes of 9 × 1, 1 × 9, and 3 × 3, respectively. Feature activation is performed using the GELU function. The resulting three feature maps are then summed together, followed by a pixel normalization operation to enhance the stability of the training process. This procedure can be formulated as follows:


(11) $$\begin{array}{l} F_{out}=Norm_{pixel}(H_{act}(Conv_{\_9\times 1}(F_{in}))+H_{act}(Conv_{\_1\times 9}(F_{in}))\\ \;+H_{act}(Conv_{\_3}(F_{in}))), \end{array}$$

where *F*_*out*_ represents the output feature after processing with the large kernel convolution, and *H*_*act*_ denotes the GELU activation function.

## 4 Experiments

In this section, the datasets, evaluation metrics and implementation details are firstly introduced in detail, and then a series of ablation experiments on ALKDNet are conducted to verify the efficiency. Next, we compare our ALKDNet with many other state-of-the art lightweight SR methods quantitatively and visually.

### 4.1 Datasets and evaluation metrics

We follow the method in previous work (Li et al., 2022) for model training and testing. DIV2K (Timofte et al., 2017) and Flickr2K (Lim et al., 2017) datasets were used for model training, and five benchmark datasets Set5 (Bevilacqua et al., 2012), Set14 (Zeyde et al., 2012), BSD100 (Arbelaez et al., 2010), Urban100 (Huang et al., 2015) and Manga109 (Matsui et al., 2017) were used for testing. LR images were generated from HR images through bicubic degradation. The evaluation of super-resolution reconstruction results is to convert the image to YCbCr format, and only calculate the PSNR and SSIM (Wang et al., 2004) of the Y component. The Multi-Adds of the evaluation method is based on the acquisition of output image with a spatial resolution of 1280 × 720 pixels.

### 4.2 Implementation details

The proposed method consists of 8 blocks and the number of channels is set to 64. The size of all convolution kernels is set to 3 unless otherwise noted. Data augmentation was performed by random rotations of 90°, 180°, 270° and horizontal flipping. The minibatch size is set to 64 and the patch size of each LR input is set to 48 × 48. We trained our model using the Adam optimizer (Kingma, 2014) with the initial learning rate set to 1 × 10^−3^, β_1_ = 0.9, β_2_ = 0.999, and adjusted the learning rate using cosine learning rate decay.*L*_1_ loss is used to optimize the model for total 1 × 10^6^ iterations. We use Pytorch 2.2.0 to implement our model on a single GeForce RTX 3090 GPU.

### 4.3 Ablation study

In this section, we demonstrate the effectiveness of the proposed method. All experiments presented here are conducted at the × 2 scaling factor.

#### 4.3.1 Impact of asymmetric large kernel convolution

We conduct ablation experiments to verify the effectiveness of the proposed large kernel convolution. We simply replaced the BSConvs used in ESDB of BSRN with the ALKConvs we designed, and explored the impact of the size of the convolution kernel on the performance. The results are shown in the Table 1. It can be found that the performance of the model has been improved when the convolution kernel size is only 5, and the comprehensive performance on each benchmark dataset has reached the best when the convolution kernel size is 9. Specifically, when the convolution kernel size is expanded to 5, the model demonstrates improved performance on all benchmark datasets except Set5 and BSD100, with an average PSNR increase of 0.04 dB and an average SSIM increase of 0.0002. Expanding the kernel size further to 9 results in an average PSNR improvement of 0.07 dB and an SSIM increase of 0.0004. We speculated that continuing to expand the convolution kernel would help further improve the performance of the model, but we decided to set the size of the convolution kernel to 9 as a trade-off between model performance and efficiency.

**Table 1:** Ablation study on large kernel convolution.

**Method**	**Params**	**Multi-adds**	**Set5**		**Set14**		**BSD100**		**Urban100**		**Manga109**	
	**(K)**	**(G)**	**PSNR**	**SSIM**	**PSNR**	**SSIM**	**PSNR**	**SSIM**	**PSNR**	**SSIM**	**PSNR**	**SSIM**
BSRN	332	73.0	38.09	0.9609	33.74	0.9193	32.24	0.9007	32.36	0.9301	39.11	0.9780
ALKConv5 × 5	354	78.3	38.09	0.9607	**33.81**	0.9197	32.24	0.9005	32.44	0.9312	39.15	0.9781
ALKConv7 × 7	361	79.9	38.11	0.9608	33.77	0.9191	32.25	0.9008	32.41	0.9307	39.20	**0.9782**
ALKConv9 × 9	368	81.6	**38.13**	**0.9610**	33.78	0.9191	**32.27**	0.9009	**32.51**	**0.9318**	**39.21**	**0.9782**
ALKConv11 × 11	375	83.2	38.08	0.9609	33.80	**0.9198**	**32.27**	**0.9010**	32.50	0.9316	39.20	0.9781

The best and second-best results are **highlighted** and underlined, respectively.

#### 4.3.2 Impact of residual learning method

In this section, we explored the impact of two residual learning methods on model performance, and the results are presented in the Table 2. Among them, FSRL is the original BSRN, ABRL is to replace the FSRL method in BSRN with ABRL, FSRL+ABRL is to add the ABRL method on the basis of the original BSRN, and with lkconv means that we replace the BSConvs in the ESDB of BSRN with our ALKConvs. It can be seen from the data in the table that the model performance has been improved after replacing the FSRL method with ABRL, but the performance decreases after applying the two residual learning methods on BSRN at the same time. However, it is interesting to see that the performance of the model is significantly improved after using large kernel convolution and two kinds of residual learning at the same time. Except for the slightly worse performance on Set5 and Set14, the best results are obtained on the other Benchmark datasets. Specifically, replacing the FSRL method with ABRL leads to an average improvement of 0.04 dB in PSNR and 0.0002 in SSIM. The highest performance is obtained when ALKConv is combined with both residual learning methods, resulting in an average gain of 0.10 dB in PSNR and 0.0006 in SSIM. On the Urban100 dataset, this method achieves a significant increase of 0.25 dB in PSNR and 0.0026 in SSIM.

**Table 2:** Ablation study on residual learning.

**Method**	**Set5**		**Set14**		**BSD100**		**Urban100**		**Manga109**	
	**PSNR**	**SSIM**	**PSNR**	**SSIM**	**PSNR**	**SSIM**	**PSNR**	**SSIM**	**PSNR**	**SSIM**
FSRL	38.09	0.9609	33.74	0.9193	32.24	0.9007	32.36	0.9301	39.11	0.9780
ABRL	38.12	0.9609	33.76	0.9194	32.24	0.9006	32.45	0.9310	39.19	0.9782
FSRL+ABRL	38.09	0.9608	33.68	0.9190	32.24	0.9006	32.40	0.9308	39.14	0.9780
ALKConv+FSRL	38.13	**0.9610**	33.78	0.9191	32.27	0.9009	32.51	0.9318	39.21	0.9782
ALKConv+ABRL	**38.14**	0.9609	**33.81**	**0.9197**	**32.28**	**0.9010**	32.49	0.9316	39.17	0.9782
ALKConv+FSRL+ABRL	38.13	0.9609	33.76	0.9192	**32.28**	**0.9010**	**32.61**	**0.9327**	**39.26**	**0.9783**

The best and second-best results are **highlighted** and underlined, respectively.

**Figure 5:**
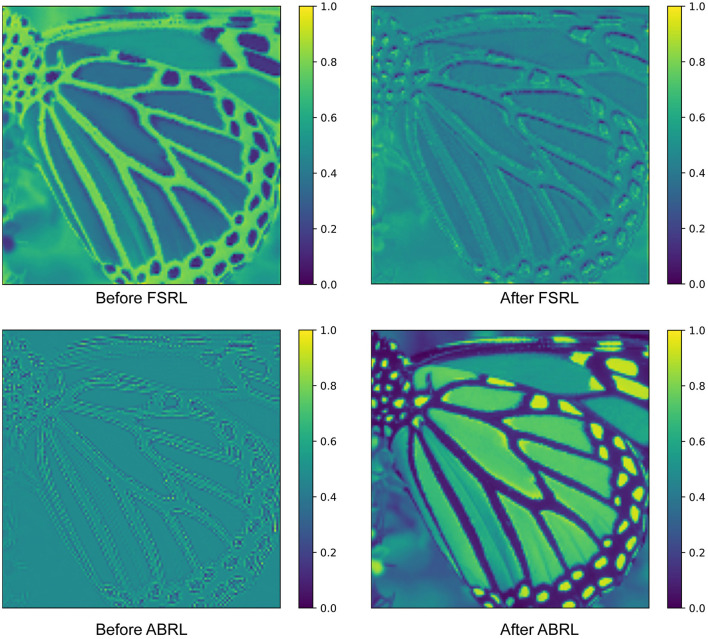
To further explore the impact of the two residual learning methods, we visualize the average feature maps obtained before and after applying FSRL and ABRL. The feature map following FSRL exhibits enhanced activation of high-frequency textures, while the feature map after ABRL contains richer detailed information.

We visualized the average feature maps before and after residual learning in Figure 5 to demonstrate the impact of residual learning. As observed, the high-frequency texture details in the feature map are effectively activated after applying FSRL. This can be attributed to FSRL's utilization of shallow features extracted by the convolutional layer for feature fusion. The convolutional layer possesses a strong capability to capture local high-frequency features, which contributes to this activation. Furthermore, after applying ABRL, the feature map exhibits a significant enhancement in image detail richness. This is primarily due to ABRL's direct utilization of information from the low-resolution image, allowing it to effectively enrich the detail representation.

#### 4.3.3 Impact of pixel normalization

In this section, we evaluate the effect of pixel normalization on model performance, as shown in Table 3. The term +norm indicates the application of pixel normalization at the end of the original ESDB. The addition of pixel normalization results in minimal impact on overall model performance, with only slight improvements observed on certain benchmarks. Specifically, incorporating the pixel normalization layer yields the greatest performance improvement on the Urban100 dataset, with an average increase of 0.05 dB in PSNR and 0.0004 in SSIM.

**Table 3:** Ablation study on pixel normalization.

**Method**	**Set5**		**Set14**		**BSD100**		**Urban100**		**Manga109**	
	**PSNR**	**SSIM**	**PSNR**	**SSIM**	**PSNR**	**SSIM**	**PSNR**	**SSIM**	**PSNR**	**SSIM**
BSRN	38.09	0.9609	33.74	0.9193	32.24	0.9007	32.36	0.9301	39.11	0.9780
BSRN+norm	38.09	0.9608	33.69	0.9189	32.25	0.9006	32.42	0.9308	39.15	0.9780
BSRN+ABRL	38.09	0.9608	33.68	0.9190	32.24	0.9006	32.40	0.9308	39.14	0.9780
BSRN+ABRL+norm	38.06	0.9607	33.72	0.9190	32.25	0.9006	32.50	0.9316	39.16	0.9781
BSRN+ALKConv	38.13	**0.9610**	33.78	0.9191	32.27	0.9009	32.51	0.9318	39.21	0.9782
BSRN+ALKConv+norm	38.13	0.9609	**33.89**	**0.9198**	32.27	0.9010	32.45	0.9313	39.21	0.9782
BSRN+ALKConv+ABRL	38.13	0.9609	33.76	0.9192	32.28	0.9010	32.61	0.9327	39.26	**0.9783**
BSRN+ALKConv+ABRL+ norm	**38.14**	0.9609	33.81	0.9193	**32.29**	**0.9011**	**32.71**	**0.9332**	**39.28**	**0.9783**

The best and second-best results are **highlighted** and underlined, respectively.

**Figure 6:**
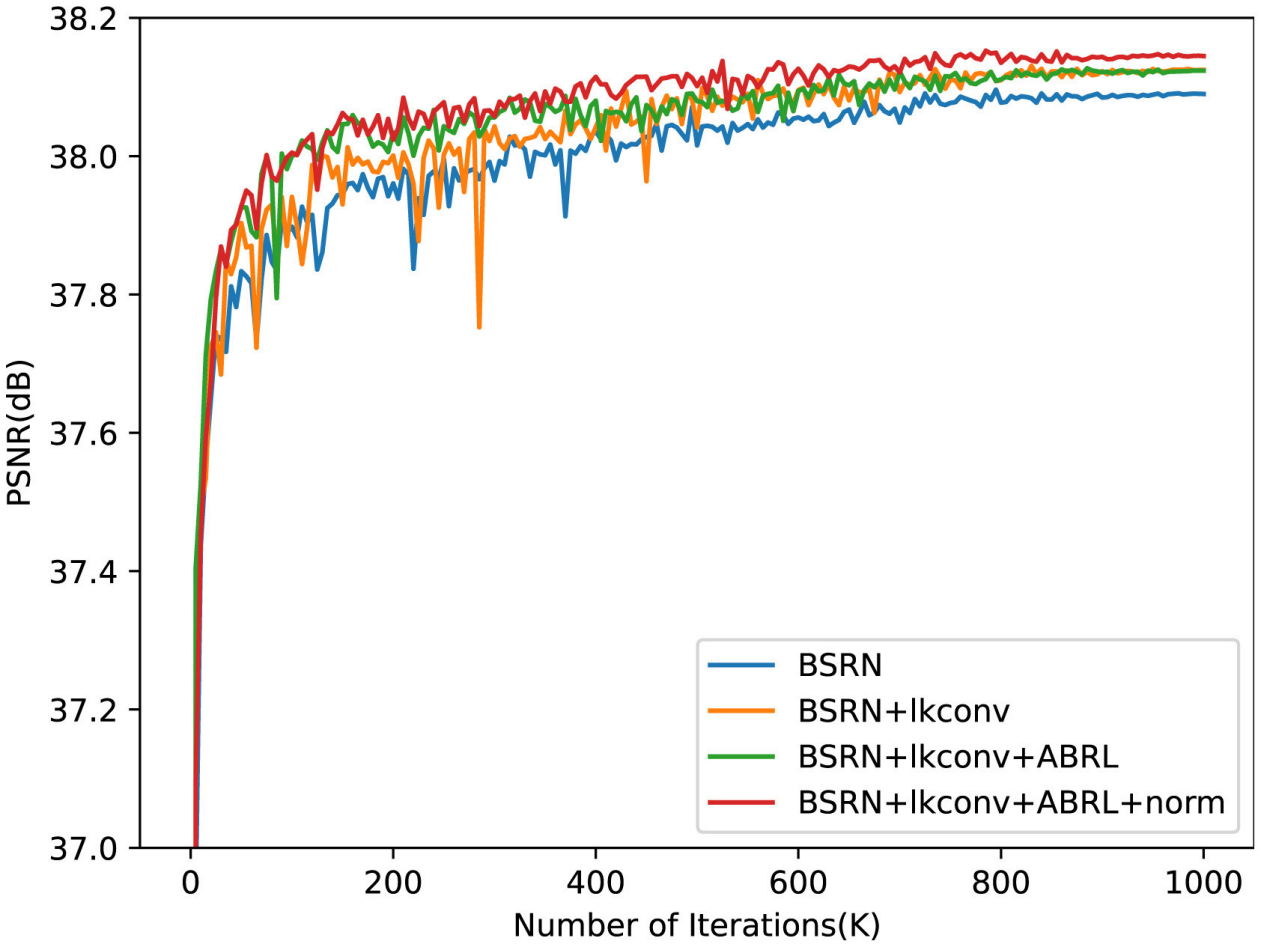
The PSNR test results on × 2 scale benchmark dataset Set5 during training. The proposed final model, as indicated by the red line, demonstrated superior performance and convergence speed, while also exhibiting the highest stability throughout the training process.

Figure 6 presents the PSNR test results during training after integrating our proposed method. The inclusion of ALKConv leads to a notable improvement in model performance, though the PSNR exhibits significant fluctuations in the early stages, suggesting instability in the training process. When ABRL is further incorporated, while the performance gain is modest, the convergence speed is notably accelerated in the initial training phase, and the overall training process becomes more stable. Finally, with the addition of pixel normalization, model performance continues to improve, and PSNR fluctuations are further reduced, indicating enhanced training stability.

### 4.4 Comparison with the state-of-the-art methods

In this section, we contrast our model with 13 other state of the art methods in lightweight SR, including SRCNN (Dong et al., 2014), FSRCNN (Dong et al., 2016), VDSR (Kim et al., 2016a), DRRN (Kim et al., 2016b), IDN (Hui et al., 2018), IMDN (Hui et al., 2019), RFDN (Liu et al., 2020), FMEN (Du et al., 2022), BSRN (Li et al., 2022), SAFMN (Sun et al., 2023), MLRN (Gendy et al., 2023), HSNet (Cui et al., 2024), and CFSR (Wu et al., 2024). Table 4 shows quantitative comparisons for × 2, × 3, and × 4 SR. It is easy to find that our model performs slightly worse on set5 of × 2 and the SSIM result is 0.0001 lower than that of BSRN, and the other test results are better than the compared advanced methods.

**Table 4:** Quantitative results of state-of-the-art lightweight SR methods on benchmark datasets.

**Method**	**Scale**	**Params**	**Multi-adds**	**Set5**	**Set14**	**BSD100**	**Urban100**	**Manga109**
		**(K)**	**(G)**	**PSNR/SSIM**	**PSNR/SSIM**	**PSNR/SSIM**	**PSNR/SSIM**	**PSNR/SSIM**
Bicubic	×2	-	-	33.66/0.9299	30.24/0.8688	29.56/0.8431	26.88/0.8403	30.80/0.9339
SRCNN	×2	8	52.7	36.66/0.9542	32.45/0.9067	31.36/0.8879	29.50/0.8946	35.60/0.9663
FSRCNN	×2	13	6.0	37.00/0.9558	32.63/0.9088	31.53/0.8920	29.88/0.9020	36.67/0.9710
VDSR	×2	666	612.6	37.53/0.9587	33.03/0.9124	31.90/0.8960	30.76/0.9140	37.22/0.9750
DRRN	×2	298	6796.9	37.74/0.9591	33.23/0.9136	32.05/0.8973	31.23/0.9188	37.88/0.9749
IDN	×2	553	124.6	37.83/0.9600	33.30/0.9148	32.08/0.8985	31.27/0.9196	38.01/0.9749
IMDN	×2	694	158.8	38.00/0.9605	33.63/0.9177	32.19/0.8996	32.17/0.9283	38.88/0.9774
RFDN	×2	534	95.0	38.05/0.9606	33.68/0.9184	32.16/0.8994	32.12/0.9278	38.88/0.9773
FMEN	×2	748	172.0	38.10/0.9609	33.75/0.9192	32.26/0.9007	32.41/0.9311	38.95/0.9778
BSRN	×2	332	73.0	38.10/**0.9610**	33.74/**0.9193**	32.24/0.9006	32.34/0.9303	39.14/0.9782
SAFMN	×2	228	52.0	38.00/0.9605	33.54/0.9177	32.16/0.8995	31.84/0.9256	38.71/0.9771
MLRN	×2	488	90.4	38.07/0.9607	33.59/0.9180	32.21/0.9000	32.28/0.9297	38.76/0.9773
HSNet	×2	302	81	38.07/0.9607	33.65/0.9185	33.22/0.9002	32.27/0.9295	39.00/0.9778
CFSR	×2	291	62.6	38.07/0.9607	33.74/0.9192	32.24/0.9005	32.28/0.9300	39.00/0.9778
ALKDNet(Ours)	×2	373	83.7	**38.14**/0.9609	**33.81**/**0.9193**	**32.29**/**0.9011**	**32.71**/**0.9332**	**39.28**/**0.9783**
Bicubic	×3	-	-	30.39/0.8682	27.55/0.7742	27.21/0.7385	24.46/0.7349	26.95/0.8556
SRCNN	×3	8	52.7	32.75/0.9090	29.30/0.8215	28.41/0.7863	26.24/0.7989	30.48/0.9117
FSRCNN	×3	13	5.0	33.18/0.9140	29.37/0.8240	28.53/0.7910	26.43/0.8080	31.10/0.9210
VDSR	×3	666	612.6	33.66/0.9213	29.77/0.8314	28.82/0.7976	27.14/0.8279	32.01/0.9340
DRRN	×3	298	6796.9	34.03/0.9244	29.96/0.8349	28.95/0.8004	27.53/0.8378	32.71/0.9379
IDN	×3	553	56.3	34.11/0.9253	29.99/0.8354	28.95/0.8013	27.42/0.8359	32.71/0.9381
IMDN	×3	703	71.5	34.36/0.9270	30.32/0.8417	29.09/0.8046	28.17/0.8519	33.61/0.9445
RFDN	×3	541	42.2	34.41/0.9273	30.34/0.8420	29.09/0.8042	28.21/0.8525	33.67/0.9449
FMEN	×3	757	77.2	34.45/0.9275	30.40/0.8435	29.17 0.8063	28.33/0.8562	33.86/0.9462
BSRN	×3	340	33.3	34.46/0.9277	30.47/0.8449	29.18/0.8068	28.39/0.8567	34.05/0.9471
SAFMN	×3	233	23.0	34.34/0.9267	30.33/0.8418	29.08/0.8048	27.95/0.8474	33.52/0.9437
MLRN	×3	496	40.9	34.46/0.9267	30.35/0.8426	29.10/0.8054	28.20/0.8533	33.66/0.9450
HSNet	×3	302	36	34.49/0.9278	30.44/0.8434	29.15/0.8063	28.36/0.8555	33.95/0.9466
CFSR	×3	298	28.5	34.50/0.9279	30.44/0.8437	29.16/0.8066	28.29/0.8553	33.85/0.9462
ALKDNet(Ours)	×3	381	37.3	**34.56**/**0.9284**	**30.50**/**0.8457**	**29.22**/**0.8079**	**28.58**/**0.8608**	**34.18**/**0.9478**
Bicubic	×4	-	-	28.42/0.8104	26.00/0.7027	25.96/0.6675	23.14/0.6577	24.89/0.7866
SRCNN	×4	8	52.7	30.48/0.8626	27.50/0.7513	26.90/0.7101	24.52/0.7221	27.58/0.8555
FSRCNN	×4	13	4.6	30.72/0.8660	27.61/0.7550	26.98/0.7150	24.62/0.7280	27.90/0.8610
VDSR	×4	666	612.6	31.35/0.8838	28.01/0.7674	27.29/0.7251	25.18/0.7524	28.83/0.8870
DRRN	×4	298	6796.9	31.68/0.8888	28.21/0.7720	27.38/0.7284	25.44/0.7638	29.45/0.8946
IDN	×4	553	32.3	31.82/0.8903	28.25/0.7730	27.41/0.7297	25.41/0.7632	29.41/0.8942
IMDN	×4	715	40.9	32.21/0.8948	28.58/0.7811	27.56/0.7353	26.04/0.7838	30.45/0.9075
RFDN	×4	550	23.9	32.24/0.8952	28.61/0.7819	27.57/0.7360	26.11/0.7858	30.58/0.9089
FMEN	×4	769	44.2	32.24/0.8955	28.70/0.7839	27.63/0.7379	26.28/0.7908	30.70/0.9107
BSRN	×4	352	19.4	32.35/0.8966	28.73/0.7847	27.65/0.7387	26.27/0.7908	30.84/0.9123
SAFMN	×4	240	14.0	32.18/0.8948	28.60/0.7813	27.58/0.7359	25.97/0.7809	30.43/0.9063
MLRN	×4	507	23.5	32.30/0.8956	28.62/0.7824	27.57/0.7365	26.10/0.7867	30.56/0.9092
HSNet	×4	313	30	32.32/0.8970	28.65/0.7838	27.63/0.7393	26.29/0.7918	30.72/0.9124
CFSR	×4	307	17.5	32.33/0.8964	28.73/0.7842	27.63/0.7381	26.21/0.7897	30.72/0.9111
ALKDNet(Ours)	×4	393	21.6	**32.37**/**0.8976**	**28.80**/**0.7860**	**27.69**/**0.7399**	**26.46**/**0.7970**	**30.97**/**0.9137**

The best and second-best results are **highlighted** and underlined, respectively.

Specifically, the performance of our model is improved compared with the suboptimal method at all three scales, for the × 2 scale, our model achieves an average improvement of 0.11 dB in PSNR and 0.0005 in SSIM. At the × 3 scale, the PSNR shows an average increase of 0.09 dB, while the SSIM improves by 0.0014. For the × 4 scale, the model delivers an average gain of 0.09 dB in PSNR and 0.0019 in SSIM. Among them, the gain of our model is the most obvious on Urban100, and the performance increases at × 2, × 3, and × 4 scales are 0.30dB/0.0021, 0.19dB/0.0041, and 0.17dB/0.0052, respectively.

**Figure 7:**
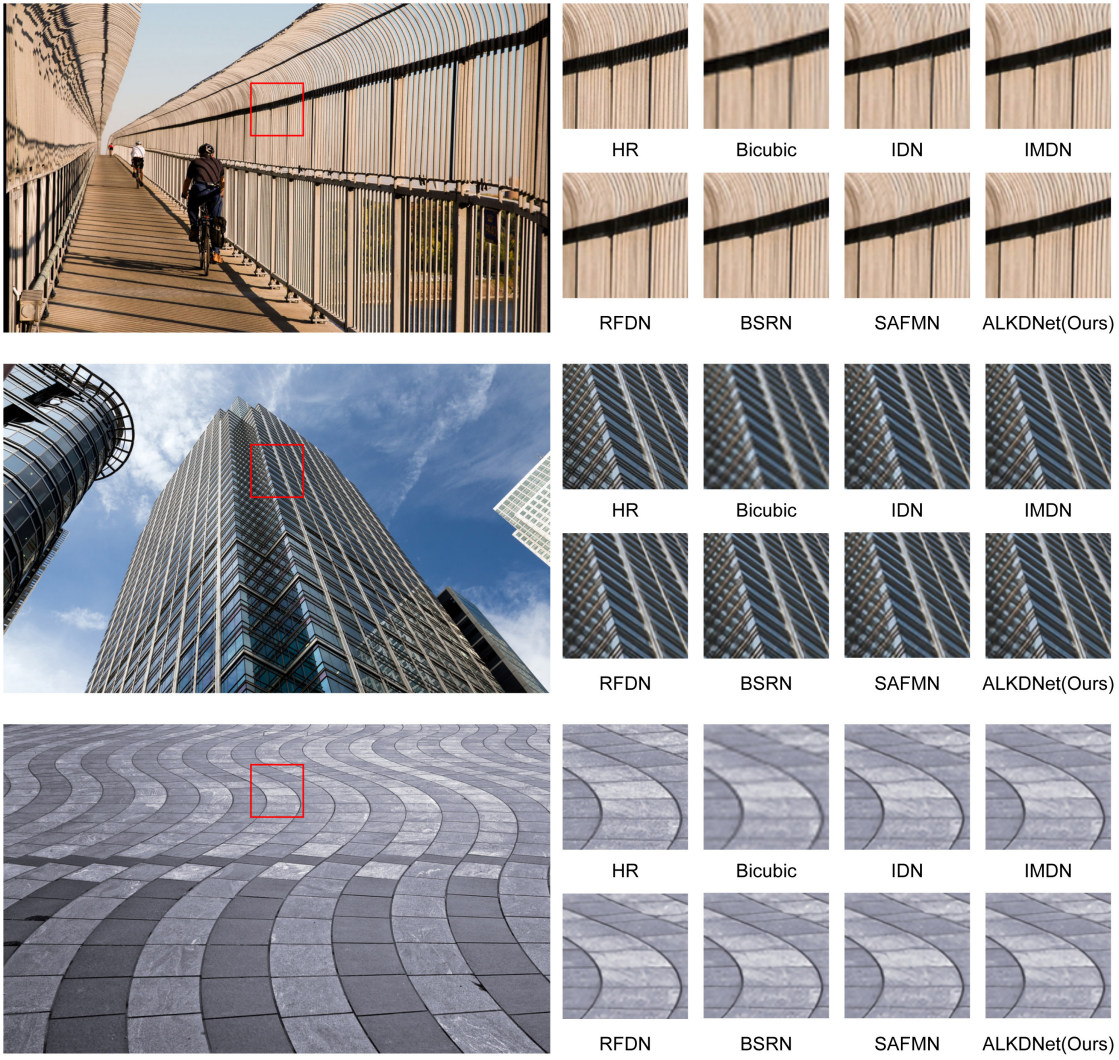
Qualitative comparison of ours model with the state-of-the-art methods for × 2 SR. We selected three images from the Urban100 dataset to evaluate and compare the visual effects of the reconstructed images. The images, presented in order from top to bottom, are img024, img047 and img071.

**Figure 8:**
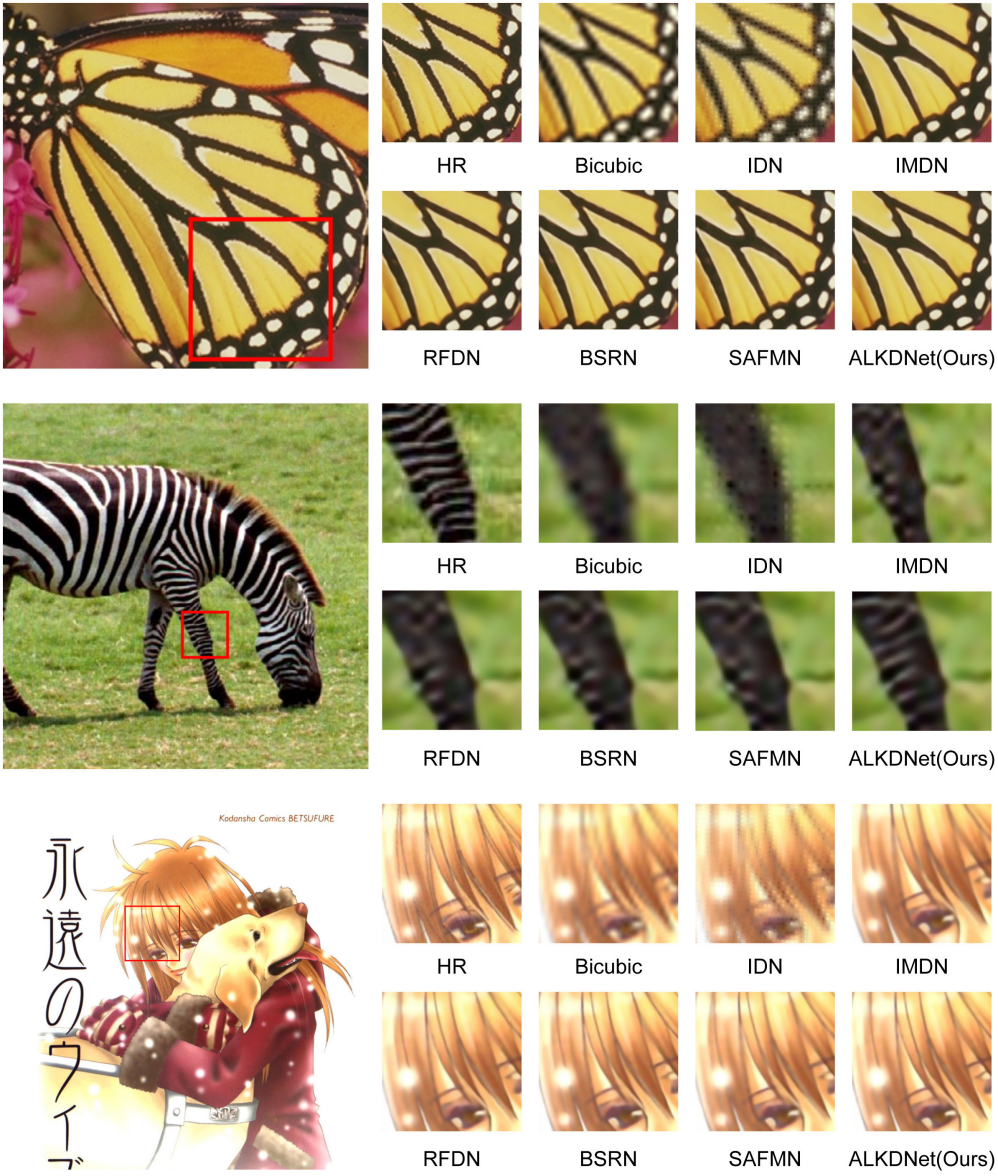
Qualitative comparison of ours model with the state-of-the-art methods for × 4 SR. We selected three images from the Set5 dataset, Set14 dataset and Manga109 dataset to evaluate and compare the visual effects of the reconstructed images. The images, presented in order from top to bottom, are butterfly in Set5, zebra in Set14 and EienNoWith in Manga109.

To demonstrate the visual effects of our model's reconstructed images, we use six images from the benchmark dataset to conduct a qualitative evaluation of the model. Figures 7, 8 displays the reconstruction results of our model compared to other state-of-the-art methods. It can be seen that our reconstruction results are still better even in the state-of-the-art methods. For example, in the image captured from img024, the images obtained by other methods have obvious artifacts at the top left continuous curved to the left texture, and the images obtained by other methods are very blurred at the bottom middle continuous vertical texture. In contrast, the image reconstructed by the proposed method is free from prominent artifacts and demonstrates the highest clarity, closely resembling the HR reference in terms of visual quality. Furthermore, within the zebra from the Set14 dataset, our method was the only one to reconstruct the high-resolution image without introducing any erroneous textures.

## 5 Conclusion

In this paper, we introduced the Asymmetric Large Kernel Distillation Network (ALKDNet), designed for lightweight super-resolution based on the BSRN architecture. The proposed method combines Asymmetric Large Kernel Convolution (ALKConv) in the distillation block, effectively balancing efficiency and performance to enhance model capability while maintaining acceptable complexity. Additionally, we introduced an anchor-point-based residual learning method in the image reconstruction module, which establishes anchor points for each corresponding pixel in the HR image using pixels from the LR image, thereby improving the quality of the reconstruction output. Results from five widely used benchmark datasets demonstrate that the proposed method achieves state-of-the-art performance.

Despite the contributions of our research, certain limitations remain. The low-resolution images used in the paper's experiments were generated through bicubic downsampling. However, in real-world scenarios, low-resolution images may be affected by various complex factors, such as limitations of acquisition devices, noise interference, and data compression. Therefore, further research is needed to effectively apply the proposed method in practical environments.

## Data Availability

The original contributions presented in the study are included in the article/supplementary material, further inquiries can be directed to the corresponding author.
